# Diffuse Capillary Malformation With Overgrowth (DCMO): A Case Report and Literature Review

**DOI:** 10.7759/cureus.35776

**Published:** 2023-03-05

**Authors:** Sarah M Fageeh, Omar S Alhothali, Sara F Alharbi, Ethar T Alsaedi, Rola R Alsulami, Ahmad N Alharbi, Ghadeer E Alamri, Khalid Al Hawsawi

**Affiliations:** 1 Medicine and Surgery, Umm Al-Qura University, Makkah, SAU; 2 Department of Dermatology, Taibah University, Medina, SAU; 3 Dermatology, King Abdulaziz Hospital Makkah, Makkah, SAU

**Keywords:** overgrowth of limb, hypertrophy, non-scaly reticulated erythematous patch, syndactyly, macrocephaly-capillary malformation (m-cm), cutis marmorata telangiectatica congenita (cmtc), vascular malformations, diffuse capillary malformation with overgrowth, capillary malformation

## Abstract

Diffuse capillary malformation with overgrowth (DCMO) is a rare condition that is characterized by capillary malformation and soft tissue hypertrophy. Here we report the case of a one-year-old male child with no past medical history who presented with skin lesions persistent since birth and associated with no symptoms. There were widespread non-scaly reticulated erythematous patches all over his body, including the abdominal wall. The circumference of the right calf and mid-thigh was 13 cm and 20 cm respectively whereas the circumference of the left calf and mid-thigh was 11 cm and 18 cm respectively. The length of both lower extremities was similar. There was also syndactyly of the right second and third toes. Differential diagnoses include cutis marmorata telangiectatica congenita (CMTC), DCMO, and macrocephaly-capillary malformation (M-CM) syndrome. Based on clinical features, the patient was diagnosed with DCMO. He was put under follow-up by pediatric orthopedics for periodic monitoring of growth asymmetry.

## Introduction

Diffuse capillary malformation with overgrowth (DCMO) was proposed in 2013 as an independent entity that is characterized by diffuse capillary malformation (CM) of the skin accompanied by proportional, nonprogressive enlargement of soft and/or bony tissue [1].

As with other vascular malformations, the cause is unknown; however, GNA11 mutation has been reported in some patients. The CM component of DCMO is characterized by widespread reticulated erythematous patches that may be confluent in some areas or appears block-like in others, often with a sharp midline demarcation on the trunk [2]. Patients with DCMO might have prominent subcutaneous veins that are not considered true venous malformation. The associated overgrowth is proportionate, not progressive, and does not correlate with the location, intensity, or morphology of the CM. Other extra-cutaneous features of DCMO include syndactyly, sandal-gap, or macrodactyly in 30% of patients [2]. Here, we report a unique case of DCMO. As the condition is rare, the patient’s presentation made his disease a diagnostic challenge.

## Case presentation

**Figure 1:**
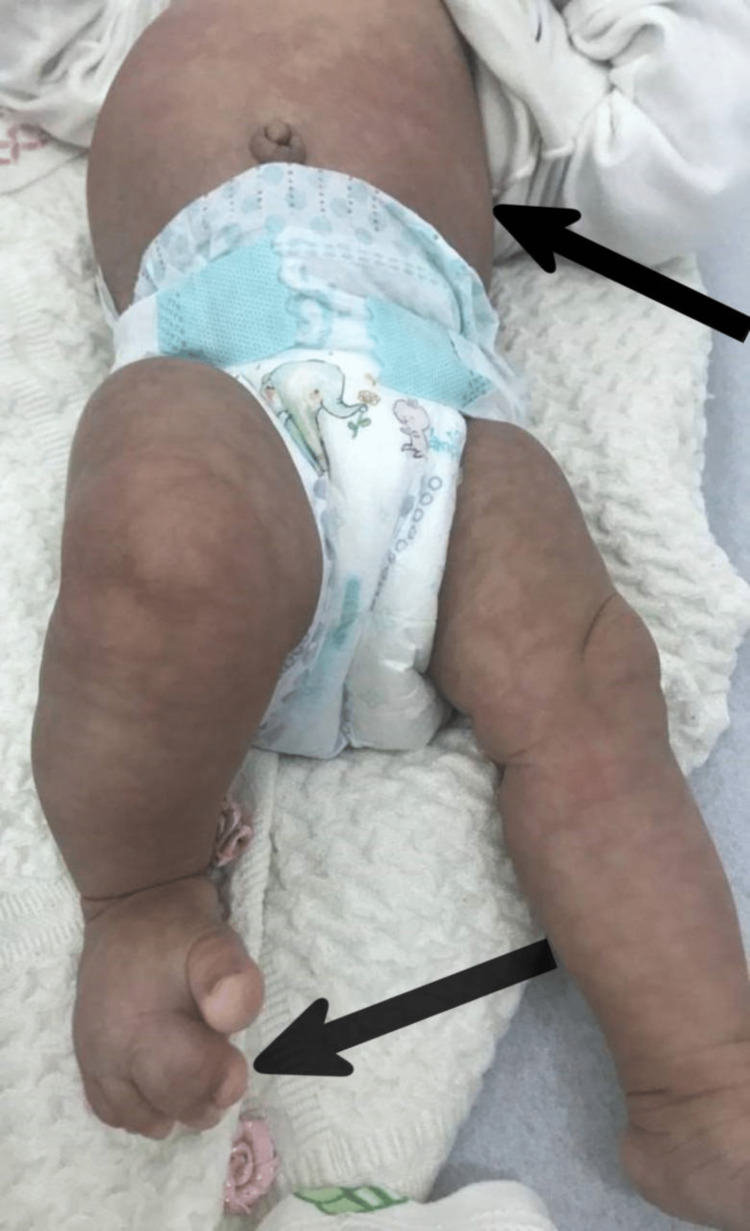
Diffuse non-scaly reticulated erythematous patches, syndactyly, and hypertrophy of the right lower limb

A one-year-old male child with no past medical history presented with skin lesions persistent since birth, not associated with any symptoms. He was born after a non-eventual full-term pregnancy and with a spontaneous vaginal delivery. There was no history of prenatal, perinatal, or postnatal complications. The systemic reviews were unremarkable. The patient was seen by a pediatric neurologist and pediatric orthopedics. There were no neurological symptoms and no family history of similar cases. Examination revealed normal head circumference. Skin examination revealed widespread non-scaly reticulated erythematous patches all over his body. There were homogenous erythematous patches on his abdominal wall too (Figure 1). Musculoskeletal examination revealed that the circumference of the right calf and mid-thigh was 13 cm and 20 cm, respectively, whereas the circumference of the left calf and mid-thigh was 11 cm and 18 cm, respectively. The length of both lower extremities was similar. There was also syndactyly of the right 2nd and 3rd toes (Figure 1). Differential diagnoses include cutis marmorata telangiectatica congenita (CMTC), DCMO, and macrocephaly-CM (M-CM) syndrome. Based on clinical features, the patient was diagnosed with DCMO. The parent was reassured and put under follow-up by pediatric orthopedics for periodic monitoring of growth asymmetry.

## Discussion

The CM in DCMO appears as pale diffuse reticulated erythema that affects more than one anatomical region [1]. Diagnosis of DCMO is sometimes confusing, especially if there are features like varicosities or skeletal anomalies as in our case. Patients can present with axial and circumferential soft tissue and bony overgrowth leading to limb length discrepancy. In comparison to Klippel-Trenaunay syndrome (KTS), which shows CM, soft tissue hypertrophy, varicosities, and skeletal anomalies, DCMO has no arteriovenous, lymphatic, or venous malformations [3]. The CM in KTS shows a geographical pattern rather than a reticulated pattern [4].

The differential diagnosis of overgrowth of the limb with the coexistence of a CM and skeletal anomalies includes KTS, CMTC, DCMO, and M-CM syndrome. Reticulated CM and syndactyly are seen in DCMO, CMTC, and M-CM syndrome; however, among these three conditions, soft tissue hypertrophy is seen only in DCMO and M-CM syndrome, whereas in CMTC, there is a limb atrophy rather than hypertrophy [5-8]. CMTC is characterized by reticulated stains, skin atrophy, and occasionally some lesions appear ulcerated [8]. Thus, the absence of macrocephaly and neurological manifestations favors the diagnosis of DCMO.

According to a retrospective review, half of the DCMO patients were wrongly diagnosed with KTS [1]. They limit the diagnosis of KTS to a limb-specific triad of a capillary-lymphatic-venous malformation (CLVM); there is no diffuse CM [1]. DCMO's variably prominent subcutaneous veins differ markedly from KTS's persisting embryologic vessels and other deep venous abnormalities [1]. Individuals with CLVM are susceptible to pulmonary embolism, cellulitis, and increasing soft tissue overgrowth throughout life. None of these conditions are observed in DCMO cases.

## Conclusions

DCMO is a newly introduced clinical diagnosis denoting a vascular anomaly, which is characterized by reticulated patches and nonprogressive soft tissue hypertrophy. It is a rare diagnosis and the presentation of the patient made his disease a diagnostic challenge. Dermatologists should be aware of its presentation as such patients require multidisciplinary management including pediatric orthopedics, neurology, and dermatology. Patients with DCMO need periodic follow-ups to monitor for leg length discrepancies.
